# Pocket contrastive learning and conformation fusion for robust structure-conditioned 3D molecular generation under receptor conformational variation

**DOI:** 10.1093/bioinformatics/btag740

**Published:** 2026-10-05

**Authors:** Huan Liu, Yijie Ding, Xiaoyi Guo, Hongjie Wu

**Affiliations:** College of Electronic and Information Engineering, Suzhou University of Science and Technology, Suzhou, 215009, China; Faculty of Computing, Harbin Institute of Technology, Harbin, 150001, China; Li Ka Shing Faculty of Medicine, The University of Hong Kong, Hong Kong SAR, China; College of Electronic and Information Engineering, Suzhou University of Science and Technology, Suzhou, 215009, China

## Abstract

**Motivation:**

Pocket-conditioned molecular generation incorporates the three-dimensional (3D) geometry and chemical environment of protein binding sites into molecular design. However, receptor conformational variation can introduce distribution shifts between training and inference, particularly under cross-docking conditions. Existing approaches often do not fully exploit the correspondence between related receptor conformations during training.

**Results:**

We propose a structure-conditioned 3D molecular generation framework that combines pocket-level contrastive learning with reference-conformation feature fusion. Built on a stepwise constructive generative model, these training-stage components encourage stable pocket representations across related conformations while preserving single-pocket inference without requiring a reference conformation. Evaluation on CrossDocked2020 and three external datasets, ASB-E, DUD-E, and APObind, demonstrates improvements in generation success, structural validity, and docking-related performance, particularly under cross-dock evaluation. These results support improved robustness of pocket-conditioned molecular generation under receptor conformational variation.

**Availability and Implementation:**

The source code and reproduction instructions are available at https://github.com/LHHHHuan/MTMGen. Pretrained models and an archival code snapshot are available at https://doi.org/10.5281/zenodo.21944857.

## 1 Introduction

In recent years, generative models have opened up new possibilities for drug discovery (Luo *et al.* 2025). Compared with traditional screening pipelines that rely on existing compound libraries, generative approaches can directly explore a much larger chemical space and propose candidate molecules under specified design objectives. In structure-based drug design, conditioning molecular generation on the three-dimensional (3D) geometry and chemical environment of a protein binding pocket enables the model to account for spatial compatibility and local interactions during generation (Jiang *et al.* 2024). This reduces the dependence on docking-only post-screening and improves the structural relevance of generated candidates.

A major challenge is that protein pockets are not static. In realistic structure-based design scenarios, related receptors or the same binding site may appear in multiple conformational states, and these conformational differences can lead to substantial changes in local geometry and interaction patterns. As a result, models trained mainly on relatively consistent structures may suffer from distribution shifts when evaluated in cross-docking settings. This issue is also relevant to receptor-conformation-aware drug design, where candidate molecules may need to remain compatible with structurally related receptor environments rather than a single fixed pocket (Anighoro *et al*. 2014).

Although large-scale structural datasets provide both re-dock and cross-dock complexes, these data are often used only as additional samples rather than as paired structural evidence. Consequently, the correspondence between different conformations within the same group is not fully exploited as an effective supervision signal. In principle, re-dock samples are usually more consistent and easier to model, whereas cross-dock samples better reflect realistic conformational variation and are therefore more challenging. An ideal model should make use of both: it should preserve the stable structural semantics learned from re-dock samples while improving robustness under the geometric discrepancies introduced by cross-docking. However, existing pipelines often mix these samples directly during training without explicitly modeling the structural relationship between them. As a result, pocket representations learned under one receptor state may transfer poorly to another, limiting cross-docking generalization.

To address this issue, we propose a structure-conditioned 3D molecular generation framework that explicitly leverages cross-conformation correspondence during training (Peng *et al*. 2022). Built on a stepwise constructive pocket-conditioned generative model, our method introduces two complementary mechanisms. First, a contrastive learning (CL) constraint on pocket-level representations encourages conformations from the same group to remain close in representation space, thereby improving robustness to receptor-state variation (Liu *et al*. 2024b). Second, for cross-dock samples, we fuse reference-conformation information to provide more stable structural context during training and reduce the impact of conformational discrepancies (Liu *et al*. 2024a, Wu *et al*. 2024a). Importantly, these enhancements are applied only during training. At inference time, the model still requires only a single target pocket as input, preserving the practicality and efficiency of the original generation interface.

Experimental results show that the proposed method improves generation success rate, structural plausibility, and docking-related metrics, indicating improved cross-docking generalization and better robustness under receptor conformational variation. Overall, this work provides a practical strategy for improving the reliability of pocket-conditioned 3D molecular generation and offers a more robust structural basis for receptor-conformation-aware drug design.

Our main contributions are summarized as follows:

1. We investigate how cross-docking data can be better exploited to improve structure-conditioned 3D molecular generation under receptor conformational variation.
2. We introduce a cross-conformation training strategy that combines pocket-level CL with reference-conformation feature fusion (FF), while keeping the original single-pocket inference interface unchanged.
3. Through experiments and metric-based evaluation, we show that the proposed method improves generation success, structural validity, and docking-related performance under cross-docking settings, providing a practical strategy for receptor-conformation-aware 3D molecular generation.

## 1.1 Related work

### 1.1.1 Sequence-based generation

Sequence-based molecular generation methods usually represent small molecules as SMILES strings or other discrete sequences, and generate candidate molecules autoregressively with models such as Transformers and their variants (Wu *et al*. 2024b). These methods benefit from abundant training data and relatively efficient training and inference, and have therefore been widely used in large-scale library expansion and property-oriented molecular optimization. To improve validity, many studies introduce grammar-based constraints, rule-based decoding, or more robust molecular string representations.

However, sequence-based methods generally do not explicitly incorporate 3D conformations or protein binding environments into the generation process. As a result, they cannot directly ensure that generated molecules are geometrically compatible with a given pocket, especially when receptor conformational changes introduce large differences in local geometry and interactions. With the increasing availability of high-quality protein structures (Jumper *et al*. 2021), research has progressively shifted from sequence-level generation toward structure-conditioned 3D molecular generation.

### 1.1.2 Structure-based conditional generation

Structure-conditioned generative methods use the local environment of a protein pocket or protein–ligand complex as input and directly generate candidate molecules in 3D space under geometric and chemical constraints (Liu *et al*. 2022, Li *et al*. 2025). Compared with sequence-based generation, these methods can better exploit pocket information and are therefore more suitable for structure-based drug design.

Recent progress in this area has been driven by structural datasets and improved geometric deep learning models. Some methods employ equivariant graph neural networks to jointly model the pocket and the growing ligand and then iteratively predict atom positions, element types, and bonding relationships (Zhang *et al*. 2023). Other methods introduce diffusion models into structure-conditioned generation, enabling molecular conformations or pocket-conditioned molecular distributions to be generated through denoising in continuous 3D space (Ho *et al.* 2020, Xu *et al.* 2022, Guan *et al.* 2023). Diffusion-based frameworks have also shown advantages in docking and conformation generation tasks (Corso *et al.* 2023).

Despite these advances, current structure-conditioned methods still face important challenges. In particular, receptor conformational variation can lead to substantial distribution shifts between training and inference, and model performance may degrade under cross-docking settings (Francoeur *et al.* 2020). These limitations have motivated further efforts toward better data utilization, stronger generalization, and more reliable evaluation protocols (Nie *et al.* 2025), especially under receptor conformational variation and cross-docking settings.

### 1.1.3 Conformation-aware generation

Protein receptors are flexible, and the same binding site may adopt different local conformations in apo, holo, or alternative ligand-bound states. These conformational changes can alter pocket shape, side-chain orientation, and key interaction patterns, making it difficult for molecules generated from one receptor state to remain compatible with another related state.

This issue is closely related to cross-docking generalization and apo–holo-aware drug design. Cross-docking evaluates ligands in non-cognate but related receptor conformations, while apo–holo-aware settings require molecules generated from ligand-free structures to remain suitable for ligand-bound conformations. Therefore, robust structure-conditioned molecular generation under receptor conformational variation remains an important and challenging problem.

Multi-target small-molecule generation aims to design compounds that can act on multiple targets simultaneously. This is important for addressing complex disease networks, improving efficacy, and reducing the risk of resistance, but it also introduces more difficult optimization constraints than single-target design. In particular, candidate molecules must balance compatibility across multiple receptor environments while also satisfying general drug-design requirements such as drug-likeness and synthesizability.

Existing studies have explored this problem from the perspective of deep generative chemistry (Ai *et al.* 2024, Munson *et al.* 2024). These works demonstrate the feasibility of using generative models for multi-target molecular design and have provided useful methodological foundations. However, the present study does not perform explicit multi-target co-optimization. Instead, it focuses on improving the robustness of single-pocket structure-conditioned 3D molecular generation under receptor conformational variation by better exploiting cross-conformation correspondence during training.

## 2 Materials and methods

### 2.1 Datasets and data preprocessing

Molecular generation under receptor conformational variation requires standardized datasets and preprocessing. We used CrossDocked2020 as the main training and internal evaluation dataset, and ASB-E, DUD-E, and APObind as external test sets.

For CrossDocked2020, we collected entries with available receptor structures and minimized ligand conformations. For each protein–ligand complex, a 10 Å binding pocket was extracted around the ligand. Specifically, a residue was retained if any of its heavy atoms was within 10 Å of any ligand heavy atom. The ligand itself was not included in the pocket file and was stored separately. Protein and ligand coordinates were kept in the same coordinate frame. Samples were removed if the receptor or ligand file was missing, the ligand could not be parsed, pocket extraction failed, or the ligand did not satisfy basic chemical validity checks.

The processed CrossDocked2020 samples were organized by group_id. Here, group_id denotes a receptor–ligand context derived from the original CrossDocked2020 organization, where related re-dock and cross-dock samples share the same ligand or pocket-level correspondence. Therefore, samples with the same group_id represent related receptor conformations or docking settings for the same binding context. During data splitting, all samples with the same group_id were assigned to the same subset to avoid direct leakage of related pocket–ligand contexts across training, validation, and testing. The final filtered CrossDocked2020 subset contained 21 066 samples, including 16 852 training samples, 2107 validation samples, and 2107 test samples, with no group_id overlap among the three subsets.

After preprocessing, each sample was represented by a protein pocket and its corresponding ligand. During training, the pocket provides the structural condition, and the ligand provides the target molecule for learning the stepwise generation process. During inference, only the target pocket is required as input, and the model generates molecules conditioned on this pocket.

The same pocket extraction and ligand preprocessing procedure was applied to ASB-E, DUD-E, and APObind. For APObind, we further performed apo–holo filtering and leakage control because it contains a relatively large number of apo–holo pairs. We retained apo–holo pairs with reliable structural correspondence using sequence identity, sequence coverage, TM-score, apo resolution, and backbone RMSD criteria. To reduce overlap with CrossDocked2020, APObind cases with overlapping apo or holo PDB identifiers were removed, and ligand-level overlap was checked using Morgan fingerprint Tanimoto similarity. This additional leakage filtering was applied only to APObind, while ASB-E and DUD-E were processed with the same basic pocket and ligand preprocessing pipeline.

### 2.2 Problem formulation

Given a protein binding pocket P, our goal is to generate a 3D small molecule M conditioned on a single input pocket while improving robustness under receptor conformational variation. The pocket is represented as a set of atoms:


(1) $$P=\{(x_{i}^{p},v_{i}^{p}){\}}_{i=1}^{N_{p}}$$

where $x_{i}^{p}\in R^{3}$denotes the 3D coordinate of the i-th pocket atom, $v_{i}^{p}\in R^{F_{p\;}}$is its atomic feature, and $N_{p}$ is the number of pocket atoms.

The molecule to be generated is similarly represented as a set of ligand atoms along with their bonding information:


(2) $$M=\{(x_{j}^{l},v_{j}^{l},b_{j}^{l}){\}}_{j=1}^{N_{l}}$$

where $x_{j}^{l}\in R^{3}$denotes the 3D coordinate of the j-th ligand atom, $v_{j}^{l}\in R^{F_{l}}$represents its atomic type features (e.g. element encoding), $b_{j}^{l}$ denotes its bonding relationships with other atoms in the molecule, and $N_{l}$ is the number of ligand atoms.

We formulate this task as learning to sample from the conditional distribution p(M∣P). The generation process follows a stepwise construction scheme. Let $M_{n}$ denote an intermediate molecular fragment containing n ligand atoms. The model ϕ iteratively expands the molecule under the pocket condition.


(3) $$M_{1}=\phi (P),\;M_{n}=\phi (M_{n-1},P),\;\;n>1$$

At each generation step, the model predicts whether to expand the current fragment and, if so, predicts the position, atom type, and bonding relationships of the next atom. During training, the pocket P and the reference ligand M are used to learn the conditional generation process. During inference, only a single target pocket is provided as input, and the model generates molecules conditioned on this pocket.

### 2.3 Generation procedure

The overall framework is illustrated in Figure 1. We aim to exploit the correspondence between re-dock and cross-dock samples within the same group to improve robustness under receptor conformational variation. Compared with relatively consistent single-conformation data, cross-dock samples contain larger geometric and interaction differences and therefore provide a more challenging training signal. To make better use of this information, we introduce two training-stage components into the stepwise pocket-conditioned generation framework: pocket-level CL and reference-conformation FF. These components are used to improve pocket representation learning during training, while the inference procedure remains conditioned on a single target pocket.

**Figure 1:**
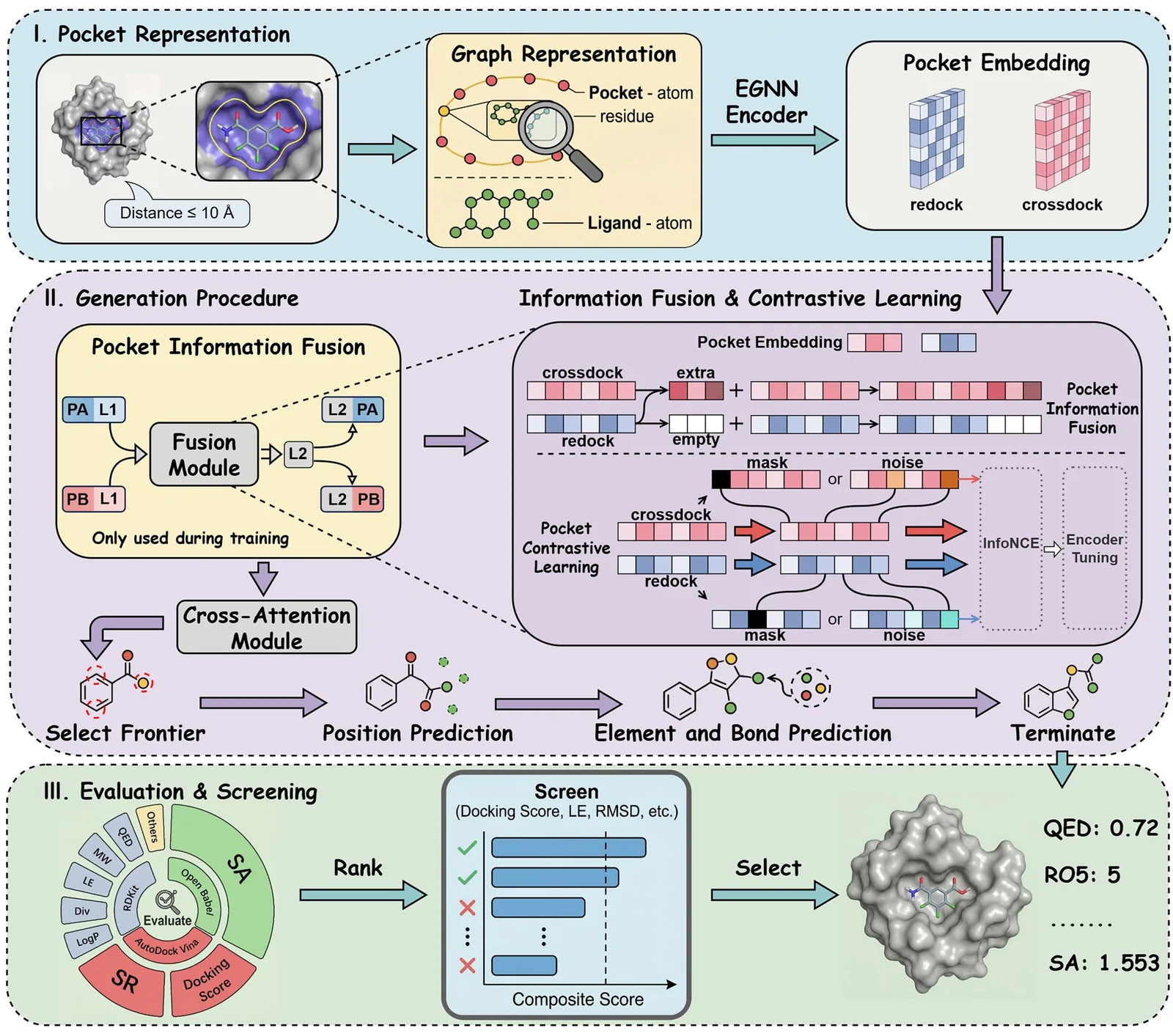
Overall architecture and workflow of the proposed method. The framework consists of three stages: pocket representation, molecular generation, and evaluation. Pocket representations are first learned from the local structural environment of the binding site under re-dock and cross-dock conditions. Molecules are then generated step by step under pocket-conditioned guidance. Finally, the generated molecules are evaluated and ranked using docking-related, geometric, and molecular-property metrics.

During generation, the model constructs the ligand structure in 3D space step by step, as illustrated in Figure 2. Given the current ligand fragment and the pocket representation, the model first identifies expandable ligand atoms and selects a focal atom. It then predicts the position of a new atom around the focal site, followed by its element type and bonding relationships with existing atoms. The newly predicted atom and its bonds are added to the current fragment, and the process is repeated until the model predicts termination or no valid expansion remains.

The contrastive constraint and reference-conformation fusion do not change the generation order. The contrastive objective encourages pocket embeddings from related conformations within the same group to learn more stable representations, whereas the reference-conformation feature provides auxiliary structural context for cross-dock samples during training. At inference time, no reference conformation is required, and the model follows the same stepwise generation procedure using only the input pocket.

**Figure 2:**
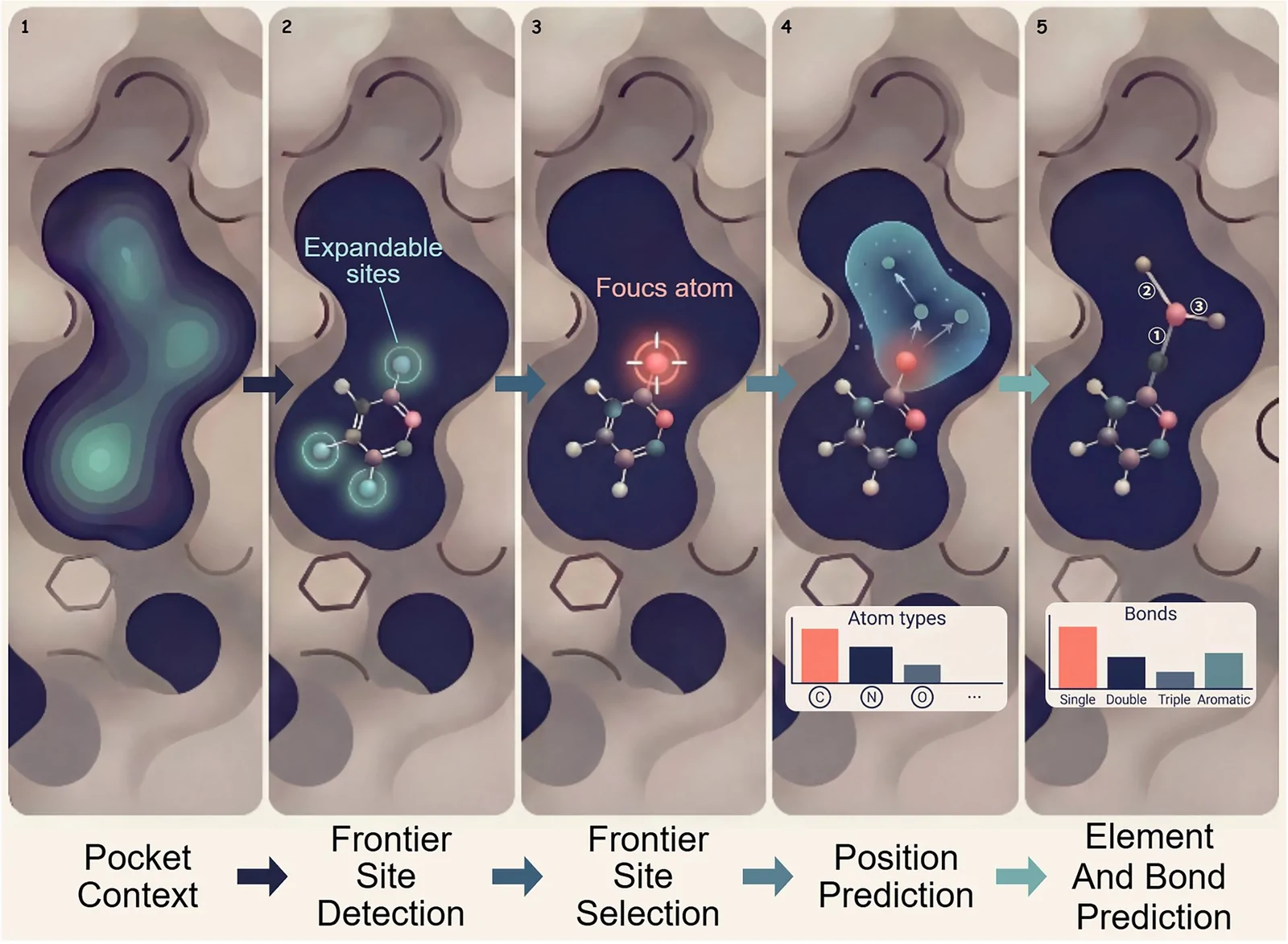
Illustration of the molecular generation process under protein pocket constraints. The model sequentially performs frontier prediction, focal-site selection, position prediction, and element/bond prediction.

### 2.4 Model architecture

Building on the generation pipeline described above, our model uses an E(3)-equivariant neural network as the backbone to stably capture geometric relationships and chemical context between the pocket and the molecular fragment in 3D space (Satorras *et al.* 2021). On top of this backbone, a joint encoder learns a unified representation of the pocket and the current ligand fragment, producing node-level and pocket-level embeddings that support subsequent generation decisions (Jing *et al.* 2021). On the decoding side, we adopt a joint predictor that, at each generation step, sequentially performs key decisions including expansion-site selection, 3D position generation, and element and bond prediction, thereby enabling stepwise constructive 3D molecular generation. The remainder of this section first introduces the E(3)-equivariant network architecture, then describes the encoder design along with the CL and information-fusion modules used for cross-dock training, and finally details the output format of the joint predictor and the coordination among its subtasks. The CL and feature-fusion modules are used only during training to improve representation robustness, while the inference procedure still follows the same single-pocket stepwise generation process.

#### 2.4.1 E(3)-equivariant neural network

The generation task in this work involves decisions that are tightly coupled with 3D geometry. Such decisions should depend only on the relative geometric relationships between the pocket and the molecular fragment, rather than on the global rotation or translation of the coordinate system. Otherwise, the same structure represented under different coordinate frames could yield inconsistent generation results, undermining the stability of cross-conformation training. To meet this requirement, we adopt an E(3)-equivariant graph neural network as the backbone, encoding the chemical and spatial context of pocket atoms and the generated fragment within a unified geometric framework (Schütt *et al.* 2017, Fuchs *et al.* 2020).

For any pair of neighboring nodes i,j, we define the relative displacement and distance as


(4) $$r_{ij}=x_{j}-x_{i},\;d_{ij}=∥r_{ij}{∥}_{2},\;{\hat{r}}_{ij}=\frac{r_{ij}}{d_{ij}+\varepsilon }$$

The distance $d_{ij}$ is expanded using Gaussian basis functions to obtain a distance embedding $g(d_{ij})$. To distinguish spatial proximity from true covalent bonds within the ligand, we further introduce a discrete bond-type feature $b_{ij}$, and concatenate it with the distance embedding to form scalar edge features:


(5) $$e_{ij}^{\text{sca}}=[g(d_{ij})\;∥\;b_{ij}\;]$$

In addition, directional information ${\hat{r}}_{ij}$ is incorporated into equivariant message passing. We apply a linear directional expansion to obtain vector-valued edge features:


(6) $$e_{ij}^{\text{vec}}=\psi ({\hat{r}}_{ij})$$

Within a single E(3)-equivariant message-passing layer, each node maintains both scalar and vector features:


$$s_{i}\in R^{d_{s}},v_{i}\in R^{d_{v}\times 3}.$$

An equivariant update operator jointly updates these features by aggregating messages from neighboring nodes j→i:


(7) $$\left(s_{i}^{\prime },v_{i}^{\prime })=F_{\theta }((s_{i},v_{i}),\{(s_{j},v_{j}),e_{ij}^{\text{sca}},e_{ij}^{\text{vec}}{\}}_{j\in N(i)}\right)$$

Here, $F_{\theta }$ denotes the aggregation and residual update process (including normalization and nonlinear transformations). By construction, this operator ensures that under any global E(3) transformation, scalar outputs remain invariant while vector features transform equivariantly.

It is important to emphasize that in this setting we do not iteratively update the node coordinates $\left.x_{i}\right.$. The coordinates are used only to construct relative geometric quantities $r_{ij}$, $d_{ij}$, and ${\hat{r}}_{ij}$, as well as the corresponding edge features.

#### 2.4.2 Joint encoder

We construct a joint graph by combining the pocket atom set P and the current ligand fragment $M_{n}$, and perform representation learning on this unified graph. Nodes in the joint graph include both pocket atoms and ligand atoms, and their geometric and edge features follow the definitions introduced in the previous section. After passing through the equivariant network, each node i in the joint graph obtains an updated representation $\left(s_{i}^{\prime },v_{i}^{\prime }\right)$.

#### 2.4.3 Pocket-level embedding

To capture global pocket semantics for contrastive alignment and reference-conformation fusion, we extract a pocket-level embedding z from the pocket node representations. Specifically, we first compute rotation-invariant scalar features from the vector channels by taking their norms, then concatenate them with the scalar features, and finally apply pooling over all pocket nodes:


(8) $$u_{i}=∥v_{i}{∥}_{2},i\in P$$

(9) $$z={\text{Pool}}_{i\in P}\;[s_{i}\;∥\;u_{i}\;]$$

Here, Pool(⋅)denotes mean pooling or an equivalent aggregation operation.

#### 2.4.4 Contrastive learning

During training, we use z to align pocket representations between re-dock and cross-dock samples within the same group. For each contrastive update, a re-dock pocket embedding is used as the anchor, and cross-dock pocket embeddings from the same group are treated as positive samples. Pocket embeddings from different groups are used as negative samples, including negatives from the same mini-batch and additional sampled negatives when needed. We optimize an InfoNCE objective:


(10) $$L_{\text{ctr}}=-\log\frac{\exp(\text{sim}(z,z^{+})/\tau )}{\exp(\text{sim}(z,z^{+})/\tau )+\sum_{k}\exp(\text{sim}(z,z_{k}^{-})/\tau )}$$

where z denotes the re-dock anchor embedding, $z^{+}$ denotes a same-group cross-dock embedding, $z_{k}^{-}$ denotes a negative embedding from a different group, sim(⋅,⋅) denotes cosine similarity, and τ is a temperature parameter.

This contrastive objective is applied at the pocket-representation level. Since the anchor and positive samples are restricted to re-dock and cross-dock pockets from the same group, the objective encourages consistency only across related receptor conformations. Meanwhile, negatives from different groups preserve inter-group discrimination and reduce the risk of collapsing different pocket semantics into a single representation.

#### 2.4.5 Feature fusion

For each cross-dock training sample, the corresponding re-dock pocket from the same group is used as the reference conformation. Its pocket embedding $z_{\text{ref}}$ is extracted and used as auxiliary structural context for the current cross-dock sample. Beyond contrastive constraints, we further incorporate reference-conformation information for cross-dock samples during training to provide a more stable structural context.

Concretely, we first map $z_{\text{ref}}$ to modulation parameters via a lightweight projection, and then apply scaling and shifting to scalar and vector node features:


(11) $$s_{i}^{'}=(1+\alpha \gamma_{s})⊙s_{i}+\alpha \beta_{s},\;v_{i}^{'}=(1+\alpha \gamma_{v})⊙v_{i}$$

where $\gamma_{s},\beta_{s}\;\mathrm{and}\;\gamma_{v}$ are produced from $z_{\text{ref}}$, and α controls the fusion strength.

This mechanism provides global structural reference information under cross-docking conditions, enabling the model to better capture shared structural semantics across conformations during expansion and prediction.

The reference information is used only during training. At inference time, no reference conformation is required; the model relies solely on the single target pocket input and follows the standard stepwise generation process.

#### 2.4.6 Joint predictor

The joint predictor takes the node representations produced by the equivariant encoder as input. During training, for cross-dock samples, these representations may include reference-conformation modulation from the fusion module. The joint predictor completes three decisions at each generation step: frontier identification, 3D position generation, and element and bond prediction. It shares the node-level representations produced by the encoder as contextual input. Let the current molecular fragment contain ligand atoms $M_{n}$, and let the selected focal atom be $f\in M_{n}$ with coordinate $x_{f}$.

#### 2.4.7 Frontier prediction

For each ligand atom $i\in M_{n}$, we first predict the probability that it is an expandable frontier site:


(12) $$p_{i}^{\text{fro}}=\sigma (f_{\text{fro}}(s_{i},v_{i}))$$

where σ(⋅) denotes the sigmoid function and $f_{\text{fro}}(\cdot )$ is the frontier prediction head. Based on $p_{i}^{\text{fro}}$, a candidate set is formed and a focal atom f is sampled.

#### 2.4.8 Position prediction

Given the selected focal atom f, the position predictor outputs a multi-modal distribution over the relative displacement $\Delta x\in R^{3}$, modeled as a Gaussian mixture:


(13) $$p(\Delta x|f)=\sum_{k=1}^{K}\pi_{k}\;N(\Delta x;\mu_{k},\Sigma_{k})$$

where $\pi_{k},\mu_{k},\Sigma_{k}=g_{\text{pos}}(s_{f},v_{f})$, $\pi_{k}$ are mixture weights, $\mu_{k}$ are means, $\Sigma_{k}$ are covariance matrices, and $g_{\text{pos}}$ is the position prediction head. After sampling Δx, the new atom coordinate is:


(14) $$x_{\text{new}}=x_{f}+\Delta x$$

#### 2.4.9 Element and bond prediction

After determining $x_{\text{new}}$, the element predictor outputs the distribution over element types:


(15) $$p(v_{\text{new}})=\text{Softmax}(f_{\text{ele}}(x_{\text{new}},\{(s_{i},v_{i},x_{i}){\}}_{i\in P\cup M_{n}}))$$

where $v_{\text{new}}$ denotes a discrete element category and $f_{\text{ele}}(\cdot )$ is the element prediction head, which can be understood as aggregating contextual features between the new atom and its environment.

Meanwhile, the bond predictor outputs the bond-type distribution between the new atom and each existing ligand atom $j\in M_{n}$:


(16) $$p(b_{\text{new},j})=\text{Softmax}(f_{\text{bond}}(x_{\text{new}},x_{j},s_{j},v_{j}))$$

Here, $b_{\text{new},j}$ denotes discrete bond types and $f_{\text{bond}}(\cdot )$ is the bond prediction head. After sampling $v_{\text{new}}$ and $\{b_{\text{new},j}{\}}_{j\in M_{n}}$, the new atom and its bonds are added to the fragment, and the model proceeds to the next generation step.

### 2.5 Training and sampling

During training, we optimize the stepwise generation objective together with the pocket-level contrastive objective to improve robustness under receptor conformational variation. Reference-conformation FF does not introduce an additional loss term; instead, it modulates the node representations of cross-dock samples during training.

#### 2.5.1 Train

The model is trained with a denoizing-recovery and stepwise construction objective. For each real ligand, we randomly mask part of the molecule and use the remaining fragment as context, allowing the model to recover the masked atoms and bonds through the same generation procedure used at inference time. The generation loss consists of ligand frontier prediction, 3D position prediction, atom-type classification, bond-type prediction, real/fake query classification, and protein-surface frontier prediction. The ligand frontier and protein-surface frontier losses are optimized with binary cross-entropy. The position loss is computed as the negative log-likelihood under a Gaussian mixture model. Atom-type and bond-type predictions are optimized with classification losses, while the real/fake query losses encourage the field predictor to distinguish valid masked atom positions from sampled negative positions. The overall training objective is:


(17) $$L_{\text{total}}=L_{\text{gen}}+\lambda_{\text{ctr}}L_{\text{ctr}}$$

(18) $$L_{\text{gen}}=L_{\text{fro}}+L_{\text{pos}}+L_{\text{cls}}+L_{\text{edge}}+L_{\text{real}}+L_{\text{fake}}+L_{\text{surf}}$$

Here, $L_{\text{fro}}$ denotes the ligand frontier prediction loss, $L_{\text{pos}}$ denotes the 3D position prediction loss, $L_{\text{cls}}$ denotes the atom-type classification loss, and $L_{\text{edge}}$ denotes the bond-type prediction loss. $L_{\text{real}}$ and $L_{\text{fake}}$ are used to distinguish real masked atom positions from sampled negative positions, and $L_{\text{surf}}$ supervises protein-surface frontier prediction. $L_{\text{ctr}}$ is the pocket-level contrastive loss, and $\lambda_{\text{ctr}}$ controls its contribution to the total objective. Reference-conformation FF is applied only to cross-dock samples during training and does not introduce a separate loss term. No reference input is required during inference.

For fair comparison, baseline models were initialized from their released official checkpoints and then further trained on the same processed CrossDocked2020 training split described in Section 2.1 whenever the training code was available. All methods used the same pocket extraction, ligand pre-processing, and train/validation/test split. During sampling and evaluation, generated molecules from all methods were processed using the same post-processing, molecular property calculation, and docking-related evaluation pipeline. This setting reduces the influence of data preprocessing and split construction, allowing a more consistent comparison across methods.

#### 2.5.2 Sample

During inference, the model takes a single target pocket as input and does not require any reference conformation. Generation starts from an initial focal site selected from the pocket, and then proceeds by iteratively expanding the growing ligand fragment. At each subsequent step, the model identifies expandable ligand atoms, selects a focal atom, predicts candidate positions for a new atom, predicts its element type, and predicts bonds between the new atom and existing ligand atoms. The new atom and its predicted bonds are then added to the fragment. Generation stops when no expandable site remains or when the predefined maximum atom number is reached. Invalid or chemically unreasonable candidates are filtered during sampling and post-processing.

## 3 Results

We organized the experimental results into three parts. First, we evaluated generation quality and pocket compatibility on four datasets, including CrossDocked2020, ASB-E, DUD-E, and APObind. CrossDocked2020 was used to assess re-dock and cross-dock performance, ASB-E and DUD-E were used as external test sets, and APObind was used to examine apo–holo generalization under receptor conformational variation. Second, we analyzed the structural plausibility of generated molecules using ring-size distributions, local geometric statistics, and representative protein–ligand visualizations. Third, we conducted ablation experiments to evaluate the contributions of pocket-level CL and reference-conformation FF.

### 3.1 Generation quality and pocket compatibility

We evaluated generation quality and pocket compatibility on four datasets: CrossDocked2020, ASB-E, DUD-E, and APObind. For each dataset, a fixed number of candidate molecules were generated for each pocket and evaluated with a unified post-processing, molecular-property, and docking-related protocol (Lipinski *et al.* 1997, Wildman and Crippen 1999, Ertl and Schuffenhauer 2009, Trott and Olson 2010, Bickerton *et al.* 2012). CrossDocked2020 was used for re-dock and cross-dock evaluation, ASB-E and DUD-E served as external test sets (Mysinger et al. 2012, Huang *et al.* 2015, Aggarwal *et al.* 2021), and APObind was used to assess apo–holo generalization under receptor conformational variation.

For all methods, generated molecules were first filtered to retain chemically valid and connected structures. The selected molecules were then docked to the corresponding full receptor structures using Vina with the same exhaustiveness and random seed. The docking box was defined according to the reference ligand. For CrossDocked2020, re-dock used the matched receptor conformation, whereas cross-dock used a paired receptor conformation from the same binding context when available. For APObind, each generated molecule was docked separately to the apo and holo receptors.

The evaluation metrics were computed as follows. Docking score was obtained from Vina. Ligand efficiency (LE) was calculated as the negative docking score divided by the number of ligand heavy atoms. Pose-adjustment RMSD was computed between the initially generated pose and the Vina-redocked pose of the same molecule using heavy atoms with the same atom order. Unless otherwise specified, the reported value refers to direct no-alignment RMSD. This metric quantifies the magnitude of pose adjustment introduced by Vina redocking rather than deviation from an experimentally determined binding pose. Therefore, a lower value indicates greater consistency between the initially generated and redocked poses, but should not be interpreted as direct evidence of higher binding-pose accuracy. SR was computed after valid-molecule filtering: a case was counted as successful if at least one selected valid molecule was successfully docked. QED, SA, RO5, and LogP were calculated as molecular-property metrics, and diversity was calculated as one minus the average pairwise molecular similarity. For datasets with two receptor settings, receptor-dependent metrics such as docking score, LE, and pose-adjustment RMSD are reported separately, whereas single-column metrics such as SR, QED, SA, RO5, diversity, and LogP are summarized as overall values.

To evaluate robustness under receptor conformational mismatch, we first tested all methods on CrossDocked2020 under both re-dock and cross-dock settings (Table 1). Our method shows clear advantages in the more challenging cross-dock setting, achieving the best cross-dock docking score and LE, the smallest pose-adjustment RMSD, and the highest SR. This indicates that the proposed training strategy improves pocket-conditioned generation when the receptor conformation changes.

**Table 1:** Quantitative results on CrossDocked2020.

Methods	Docking score (↓)		Ligand efficiency (↑)		RMSD (↓)		SR (↑)	QED (↑)	SA (↓)	RO5 (↑)	Diversity (↑)	LogP
	Re-dock	Cross-dock	Re-dock	Cross-dock	Re-dock	Cross-dock						
ED2Mol	−6.340	−6.287	0.435	0.432	5.303	5.665	0.9939	0.7220	1.9706	4.9995	0.8635	2.0096
ResGen	−6.614	−6.536	0.386	0.382	6.410	6.605	0.8076	0.5578	2.1242	4.8279	0.8087	1.5891
TargetDiff	−7.399	−7.159	0.265	0.257	5.961	6.837	0.7722	0.3595	3.2715	4.2559	0.8869	1.8469
Pocket2Mol	−6.629	−6.475	0.442	0.433	6.234	6.231	0.8995	0.5439	1.9885	4.9670	0.8270	1.5547
GraphBP	−6.117	−6.064	0.428	0.424	12.278	12.436	0.9914	0.5050	2.4720	4.8407	0.9023	3.1052
Apo2Mol	−7.083	−6.983	0.320	0.317	6.800	7.177	0.9414	0.4664	4.7064	4.3505	0.8727	3.4807
Ours	−7.367	−7.238	0.437	0.434	5.453	5.538	0.9943	0.5983	1.9504	4.9802	0.9098	1.7732

In the cross-dock setting, our method achieves the most favorable docking score, the highest ligand efficiency, and the lowest RMSD among the compared methods. It also achieves the highest success rate (SR). RMSD (Å) is calculated between the initially generated and Vina-redocked poses without rigid-body alignment.

We then evaluated transferability on ASB-E and DUD-E (Tables 2 and 3). On ASB-E, our method achieves the best LE, the smallest pose adjustment during Vina redocking, and the highest SR, showing favorable docking-related behavior under the adopted evaluation protocol. On DUD-E, it also obtains the highest SR and maintains competitive docking-related performance. Although some baselines perform better on individual molecular-property metrics, our method shows more balanced overall behavior.

**Table 2:** Quantitative results on ASB-E.

Methods	Docking score (↓)	Ligand efficiency (↑)	RMSD (↓)	SR (↑)	QED (↑)	SA (↓)	RO5 (↑)	Diversity (↑)	LogP
ED2Mol	−6.258	0.4472	0.9138	0.9643	0.7098	1.5628	4.9927	0.8318	1.9150
ResGen	−6.530	0.4493	0.8827	0.9080	0.5661	1.5513	4.8703	0.8152	1.1989
TargetDiff	−7.697	0.2731	1.7828	0.7033	0.3611	2.4168	4.2742	0.8889	1.8613
Pocket2Mol	−6.360	0.4447	0.9043	0.9216	0.5153	1.5667	4.9990	0.8585	0.5690
GraphBP	−5.972	0.4365	1.1670	0.8711	0.4956	1.9853	4.8393	0.9038	3.1088
Apo2Mol	−7.006	0.3453	1.5260	0.8609	0.4308	4.7203	4.3131	0.8864	2.8271
Ours	−7.051	0.4587	0.8762	0.9916	0.5463	1.5418	4.8458	0.8465	1.2786

Our method achieves the highest ligand efficiency and success rate (SR), together with the lowest aligned RMSD among the compared methods. RMSD (Å) is calculated after rigid-body alignment of the initially generated and Vina-redocked poses.

**Table 3:** Quantitative results on DUD-E.

Methods	Docking score (↓)	Ligand efficiency (↑)	RMSD (↓)	SR (↑)	QED (↑)	SA (↓)	RO5 (↑)	Diversity (↑)	LogP
ED2Mol	−6.850	0.4678	0.9437	0.9703	0.7257	1.5816	4.9991	0.8085	2.0083
ResGen	−7.692	0.3798	1.0124	0.8600	0.5517	1.6979	4.7084	0.8049	2.5317
TargetDiff	−8.799	0.2839	1.7102	0.7937	0.3790	2.4360	4.2672	0.8888	2.6068
Pocket2Mol	−6.524	0.4720	0.9239	0.7773	0.5410	1.5234	4.9997	0.8636	0.9112
GraphBP	−6.507	0.4628	1.1770	0.8657	0.4970	1.9852	4.8537	0.9022	2.9960
Apo2Mol	−7.7576	0.3011	1.8520	0.951	0.428	4.9268	4.0354	0.878	4.6217
Ours	−8.872	0.3929	1.1673	0.9802	0.5952	1.6921	4.8099	0.8300	2.8262

Our method achieves the most favorable docking score and the highest success rate (SR) among the compared methods. RMSD (Å) is calculated after rigid-body alignment of the initially generated and Vina-redocked poses.

To further assess apo–holo robustness, we evaluated all methods on APObind, where each case contains paired apo and holo receptors (Table 4). Our method performs consistently in both receptor states and achieves the most favorable holo docking score and LE, the smallest holo pose-adjustment RMSD, and the highest SR. Compared with Apo2Mol (Zheng *et al.* 2026), our method remains competitive while requiring only a single target pocket during inference.

**Table 4:** Quantitative results on APObind.

Methods	Docking score (↓)		Ligand efficiency (↑)		RMSD (↓)		SR (↑)	QED (↑)	SA (↓)	RO5 (↑)	Diversity (↑)	LogP
	Apo	Holo	Apo	Holo	Apo	Holo						
ED2Mol	−5.908	−6.416	0.412	0.448	5.975	5.954	0.9906	0.7164	7.1626	4.9996	0.8393	1.9886
ResGen	−5.774	−6.654	0.387	0.442	7.374	6.844	0.8434	0.5745	2.9161	4.9376	0.8166	1.5722
TargetDiff	−6.707	−7.494	0.229	0.257	6.799	6.914	0.8312	0.3246	5.0382	3.9400	0.8843	2.4857
Pocket2Mol	−6.062	−6.823	0.393	0.444	5.999	5.765	0.9976	0.5863	2.8916	4.9666	0.8071	1.2103
GraphBP	−5.110	−5.604	0.432	0.433	13.608	13.294	0.9835	0.4521	4.6495	4.8808	0.9119	2.0600
Apo2Mol	−6.693	−7.481	0.269	0.303	7.769	7.391	0.9789	0.4574	4.8491	4.1473	0.8778	4.5152
Ours	−6.795	−7.692	0.415	0.475	5.983	5.716	0.9983	0.6159	2.8991	4.8318	0.9183	1.4822

Our method achieves the most favorable docking score and the highest ligand efficiency in both apo and holo settings, together with the lowest holo RMSD and the highest success rate (SR). RMSD (Å) compares the initially generated pose with the pose obtained by Vina redocking to the corresponding apo or holo receptor.

The main quantitative results are further visualized in Figs 3–5. Figure 3 summarizes the normalized multi-metric comparison across the four datasets. Figure 4 compares pose-adjustment RMSD distributions on ASB-E and DUD-E, showing that our method has relatively concentrated distributions and fewer cases requiring large adjustments during Vina redocking. Figure 5 shows APObind apo–holo docking score distributions, further supporting the robustness of our method under receptor conformational changes.

**Figure 3:**
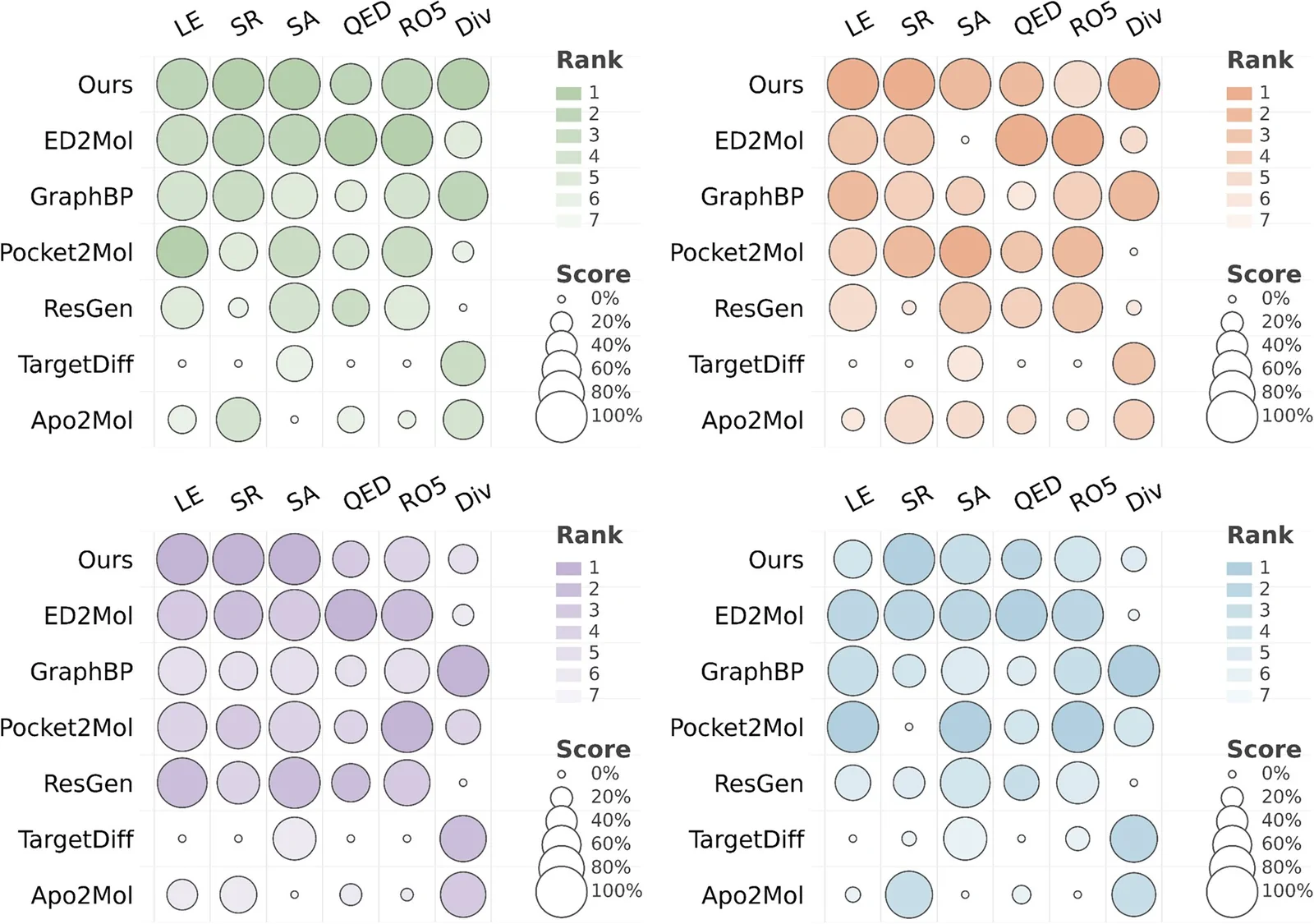
Normalized multi-metric comparison across four datasets. Panels a–d correspond to CrossDocked2020, APObind, ASB-E, and DUD-E, respectively.

**Figure 4:**
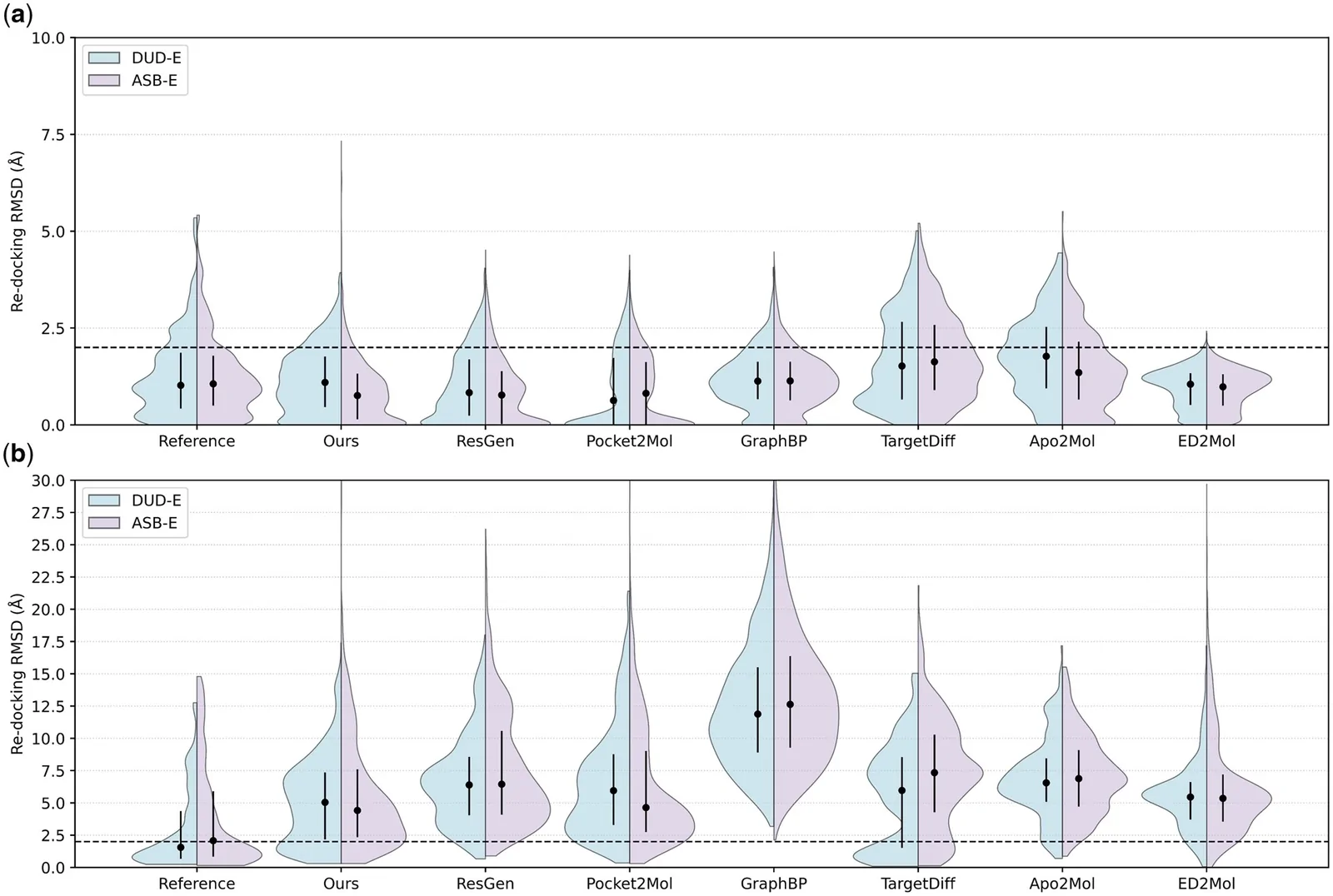
Pose-adjustment RMSD distributions on ASB-E and DUD-E. Panels a and b show aligned and no-alignment pose-adjustment RMSD, respectively.

**Figure 5:**
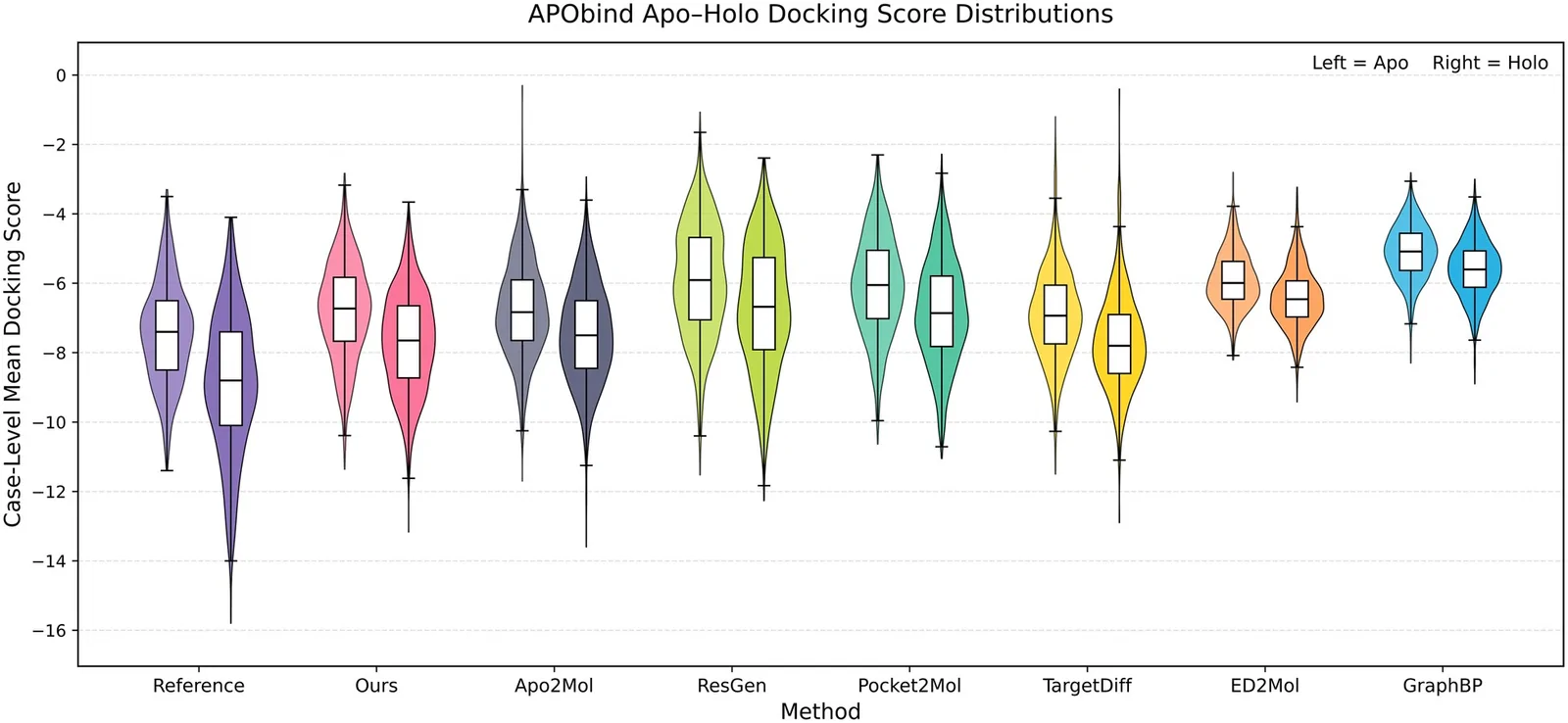
APObind apo–holo docking score distributions.

### 3.2 Molecular geometry and substructure analysis

Beyond docking and molecular-property metrics, we further examined whether the generated molecules preserve reasonable substructure and local geometry. This analysis was conducted on ASB-E and DUD-E, the two external test sets used in Section 3.1.

We first compared ring-size distributions between generated molecules and test-set molecules (Fig. 6). Ring systems are important drug-like substructures, and their size distribution reflects whether a method generates realistic molecular scaffolds. On both datasets, our method follows the major test-set pattern, mainly producing common five- and six-membered rings, while avoiding excessive generation of uncommon ring sizes.

**Figure 6:**
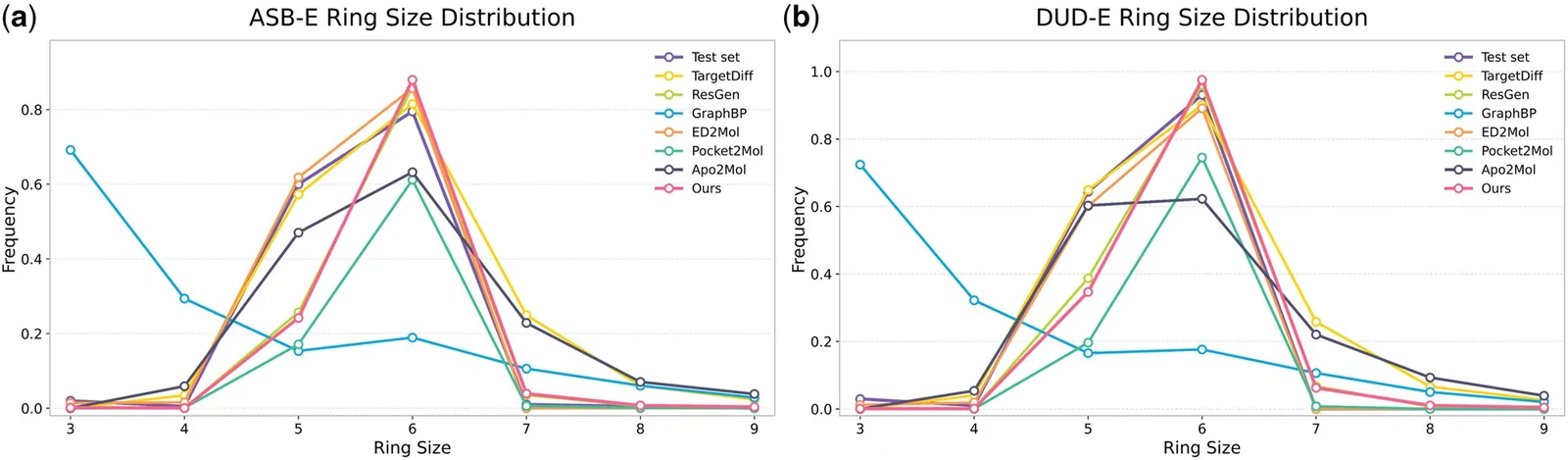
Ring-size distributions on ASB-E and DUD-E.

We then evaluated local molecular geometry using bond-angle and dihedral-angle statistics (Fig. 7). KL divergence was used to compare generated local geometric distributions with those of reference molecules. Our method generally shows low KL divergence across multiple angle and dihedral patterns on both datasets, indicating stable local geometry without obvious outlier deviations.

**Figure 7:**
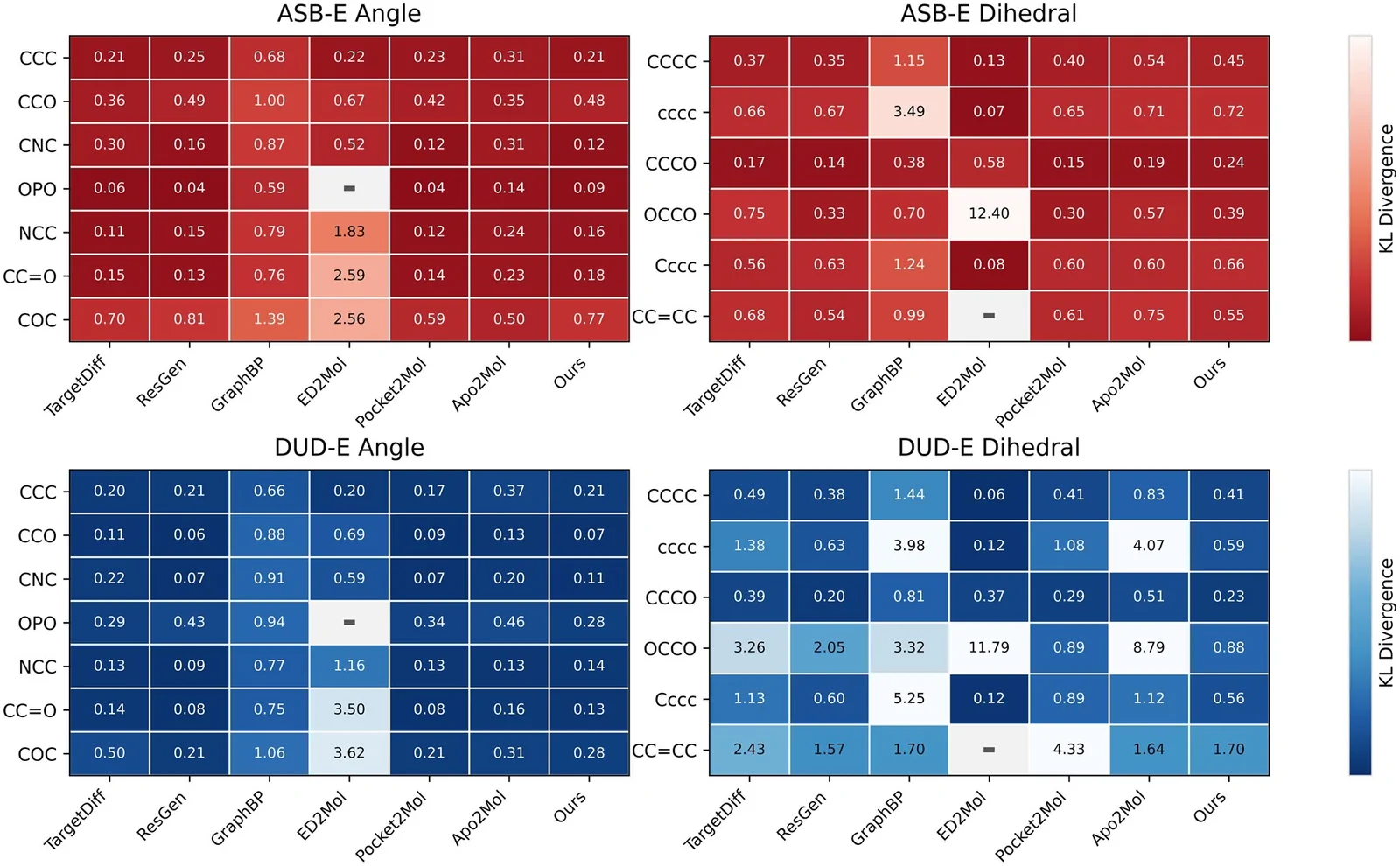
KL divergence of local angle and dihedral distributions.

Finally, we visualized several generated molecules in protein binding pockets using PyMOL (Fig. 8). These examples were randomly selected from successfully docked molecules with favorable Vina docking scores in the quantitative evaluation, rather than manually designed for visualization. The generated molecules can occupy the binding pocket and form spatially plausible poses, complementing the quantitative results with qualitative pocket-level evidence.

**Figure 8:**
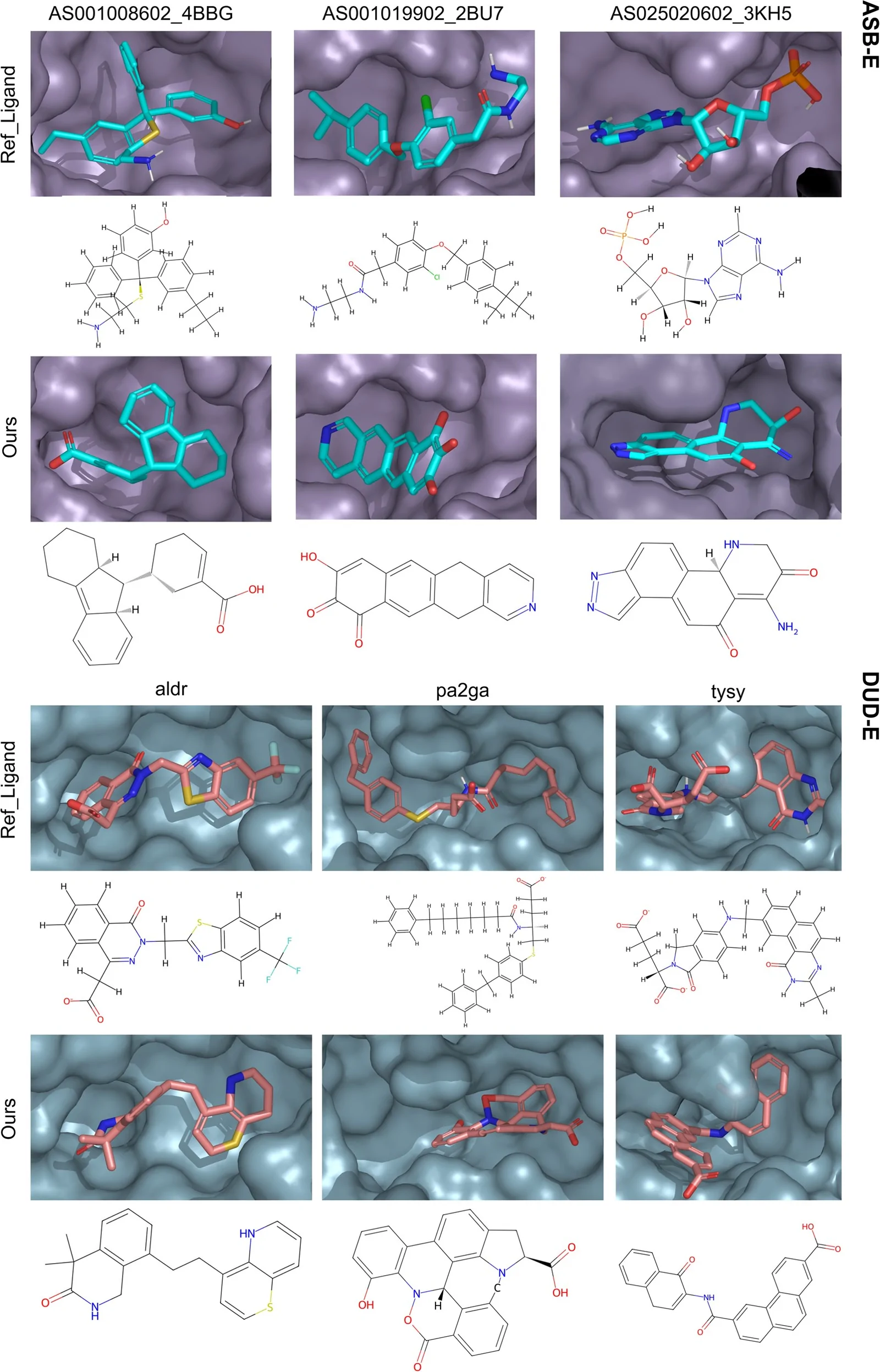
Examples of generated molecules in protein binding pockets.

### 3.3 Ablation study

To evaluate the contribution of pocket-level CL and reference-conformation FF, we performed an ablation study by removing each component from the full model (Table 5). The full model with both CL and FF achieves the best overall performance, including the most favorable docking score, the highest LE, the smallest pose-adjustment RMSD, and the highest SR. These results indicate that the two components jointly improve the docking-related metrics and the consistency between initially generated and Vina-redocked poses under the adopted evaluation protocol. Removing either CL or FF weakens the docking-related metrics. When CL is removed, the docking score changes from −7.303 to −6.986, pose-adjustment RMSD increases from 5.495 to 5.683, and SR decreases from 0.9943 to 0.9437. When FF is removed, LE decreases from 0.436 to 0.416, RMSD increases to 5.661, and SR decreases to 0.9141. These results suggest that CL helps stabilize pocket representations across related receptor conformations, while FF provides useful auxiliary structural context during training. The largest performance drop is observed when both components are removed. In this setting, the docking score, LE, RMSD, and SR all become worse than those of the full model. This confirms that CL and FF are complementary rather than redundant. In contrast, molecular-property metrics such as QED, SA, RO5, diversity, and LogP show only moderate changes across ablation variants. This is expected because CL and FF mainly affect pocket representation learning and cross-conformation robustness, rather than directly optimizing general molecular-property distributions.

**Table 5:** Ablation study of contrastive learning and feature fusion.

Methods	Docking score (↓)	Ligand efficiency (↑)	RMSD (↓)	SR (↑)	QED (↑)	SA (↓)	RO5 (↑)	Diversity (↑)	LogP
CL + FF	−7.303	0.436	5.495	0.9943	0.5983	1.9504	4.9802	0.9098	1.773
w/o CL	−6.986	0.427	5.683	0.9437	0.5832	1.9578	4.9742	0.8907	1.669
w/o FF	−7.029	0.416	5.661	0.9141	0.5824	1.9581	4.9708	0.9016	1.767
w/o Both	−6.556	0.410	5.842	0.8938	0.5493	1.9658	4.9620	0.8925	1.564

CL denotes pocket-level contrastive learning, and FF denotes reference-conformation feature fusion. The full model (CL + FF) achieves the most favorable docking score, the highest ligand efficiency and success rate (SR), and the lowest RMSD among the evaluated variants, supporting the complementary contributions of the two components.

## 4 Discussion

Pocket-conditioned 3D molecular generation is important for structure-based drug design, but robust generation under receptor conformational variation remains challenging. In this work, we exploit the cross-conformation correspondence between re-dock and cross-dock samples through pocket-level CL and reference-conformation FF. The results show that this strategy improves docking-related metrics and the consistency between initially generated and Vina-redocked poses under receptor variation, while preserving the single-pocket inference setting.

The main advantage of our framework is that it uses cross-docking information as structured supervision, rather than treating all complexes as independent training samples. Pocket-level CL helps stabilize pocket representations across related receptor conformations, while reference-conformation FF provides additional structural context for cross-dock samples. Therefore, the model can better adapt to receptor-state changes without requiring additional receptor conformations during inference.

Several observations from the experiments are worth noting. First, ED2Mol shows clear advantages in molecular-property metrics such as QED and RO5, suggesting that its generation strategy may better preserve drug-like and chemically favorable property distributions. In contrast, the main advantage of our method lies in docking robustness under receptor conformational variation, rather than outperforming all baselines on every molecular-property metric. Second, Apo2Mol is designed for apo–holo-aware generation and can model pocket changes together with ligand generation. In our evaluation, however, its generated ligands were docked back to fixed apo and holo receptor structures for a unified comparison, which may not fully reflect the compatibility between Apo2Mol-generated ligands and its induced pocket conformations. Third, different methods show different tendencies in molecule size and sampling behavior. Earlier methods such as GraphBP and Pocket2Mol tend to generate smaller molecules in our experiments, whereas diffusion-related methods more often generate larger molecules but may require higher sampling cost. This phenomenon may be related to model architecture, atom-count priors, generation order, stopping criteria, and post-processing strategies, and requires further analysis.

Several limitations remain. The current evaluation mainly relies on computational metrics, including molecular properties, docking scores, and geometric statistics, which cannot fully represent true binding affinity or biological activity. Importantly, the pose-adjustment RMSD used in this study does not compare generated poses with experimentally determined ligand poses and therefore should not be interpreted as a direct measure of binding-pose accuracy. CrossDocked2020 also contains docking-generated complexes and may introduce docking-protocol-specific bias. Although external test sets and APObind apo–holo evaluation help assess generalization, the results should still be interpreted as computational evidence. In addition, the proposed method improves robustness under receptor conformational variation but does not perform explicit multi-target co-optimization.

Future work may further strengthen molecular-property control to better balance docking robustness with QED-, RO5-, and SA-related properties. More explicit receptor-flexibility modeling could also be introduced so that ligand generation is not limited to a fixed-pocket assumption. In addition, systematic analysis of molecule-size control, sampling efficiency, and different generation paradigms will be useful for understanding their effects on downstream docking behavior. Incorporating richer physical interaction modeling and experimental validation will further improve the reliability of such models in practical drug discovery.

## 5 Conclusion

Overall, this work provides a practical strategy for improving the robustness of pocket-conditioned 3D molecular generation by leveraging cross-conformation correspondence during training while keeping inference conditioned on a single target pocket.

## Data Availability

The source code and instructions for reproducing the experiments are publicly available at https://github.com/LHHHHuan/MTMGen. The pretrained models and an archival snapshot of the code corresponding to the results reported in this article are available on Zenodo at https://doi.org/10.5281/zenodo.21944857. The datasets analyzed in this study are publicly available from the original sources cited in Section 2.1.
